# A Risk Explicit Interval Linear Programming Model for Uncertainty-Based Environmental Economic Optimization in the Lake Fuxian Watershed, China

**DOI:** 10.1155/2013/824078

**Published:** 2013-09-26

**Authors:** Xiaoling Zhang, Kai Huang, Rui Zou, Yong Liu, Yajuan Yu

**Affiliations:** ^1^College of Environmental Science and Engineering, Beijing Forestry University, Beijing 100083, China; ^2^Tetra Tech, Inc. 10306 Eaton Place, Ste 340, Fairfax, VA 22030, USA; ^3^College of Environmental Science and Engineering, Key Laboratory of Water and Sediment Sciences (MOE), Peking University, Beijing 100871, China; ^4^Beijing Key Laboratory of Environmental Science and Engineering, School of Chemical Engineering & Environment, Beijing Institute of Technology, Beijing 100081, China

## Abstract

The conflict of water environment protection and economic development has brought severe water pollution and restricted the sustainable development in the watershed. A risk explicit interval linear programming (REILP) method was used to solve integrated watershed environmental-economic optimization problem. Interval linear programming (ILP) and REILP models for uncertainty-based environmental economic optimization at the watershed scale were developed for the management of Lake Fuxian watershed, China. Scenario analysis was introduced into model solution process to ensure the practicality and operability of optimization schemes. Decision makers' preferences for risk levels can be expressed through inputting different discrete aspiration level values into the REILP model in three periods under two scenarios. Through balancing the optimal system returns and corresponding system risks, decision makers can develop an efficient industrial restructuring scheme based directly on the window of “low risk and high return efficiency” in the trade-off curve. The representative schemes at the turning points of two scenarios were interpreted and compared to identify a preferable planning alternative, which has the relatively low risks and nearly maximum benefits. This study provides new insights and proposes a tool, which was REILP, for decision makers to develop an effectively environmental economic optimization scheme in integrated watershed management.

## 1. Introduction

Watershed has been a highly preferable study unit combining terrestrial with aquatic systems since watershed programming was proposed in the 1970s for the first time [1, 2]. With the rapid economic development within watersheds, human activity has caused severely adverse consequences on natural surface water bodies, such as water resources shortage [3], water quality deterioration [4], and aquatic ecological degradation [5]. At the same time, surface water bodies' deterioration has inversely become the bottleneck of restricting the sustainable development of watershed [6]. The conflicting relationship between economic benefit and environmental protection should be harmonized at the watershed scale, and water environment and socioeconomic systems should be integrated into account in watershed management [7].

In the integrated watershed systems, environmental protection, rational resources utilization, and sustainable economic development are three principal management goals which strongly depend on the structure and development of three main industries. At present, economic development could be efficiently encouraged by optimizing industrial structure [8]. However, the primary industrial development may bring widespread nonpoint pollution, such as farmland runoff pollution, rural domestic sewage, and pollution of livestock and poultry breeding. The secondary industry could then cause serious point pollution, for example, sewage discharge from factories and urban families. Therefore, industrial structure is also a key linkage of economic benefit to ecological environmental system. Unreasonable industrial structure is one of the main reasons for excessive anthropogenic nutrient inputs to aquatic ecosystems [9]. Above all, industrial structure must be optimized to control extensive watershed nutrient loading, especially nonpoint source (NPS) loading to meet the requirements of environmental capacity.

Mathematical programming has been comprehensively applied in environmental economic optimization. Stochastic programming (SP), fuzzy programming (FP), and interval programming (IP) [10–12] are dominant approaches to consider various uncertainties of watershed systems, which derive from the randomness of natural process and sampling data, the vagueness of model structure, coefficients and equations, and lack of professional and background knowledge [13], among which interval linear programming (ILP) is the best choice for practical optimization problems under uncertainties because model parameters could be easily estimated by the lower and upper bounds [14]. However, two severe limitations of traditional ILP in decision making have been found in recent researches: (1) the optimal solutions of ILP may lead to invalid and infeasible optimization schemes [15]; (2) ILP could not make trade-off between risk and system return [16]. In 2010, Zou et al. [17] put forward a risk explicit interval linear programming (REILP) approach to overcome the shortcomings of ILP solutions. After that, the REILP-based watershed nutrient-load reduction model was applied in Lake Qionghai watershed and proved effective in formulating implementation schemes [16].

The objective of this paper is to explore the applicability of REILP approach for watershed environmental economic optimization in the Lake Fuxian watershed, China. Lake Fuxian is the second deepest freshwater lake in China. Previous studies showed that industrial structure in Lake Fuxian watershed was extremely unreasonable and resulted in severe water pollution problems [18]. Therefore, comprehensive planning of industrial structure was crucial for water environment protection and watershed sustainable development. This paper applied the REILP model to balance the optimal system returns and uncertainty risks with the consideration of decision makers' preferences so as to develop an efficient industrial restructuring scheme in Lake Fuxian watershed. The findings of this study provide new insights and a tool for policy makers to develop environmental economic optimization scheme of their regions, especially regions with severe conflict of water environment protection and economic development.

## 2. Materials and Methodology

### 2.1. Study Area

**Figure 1:**
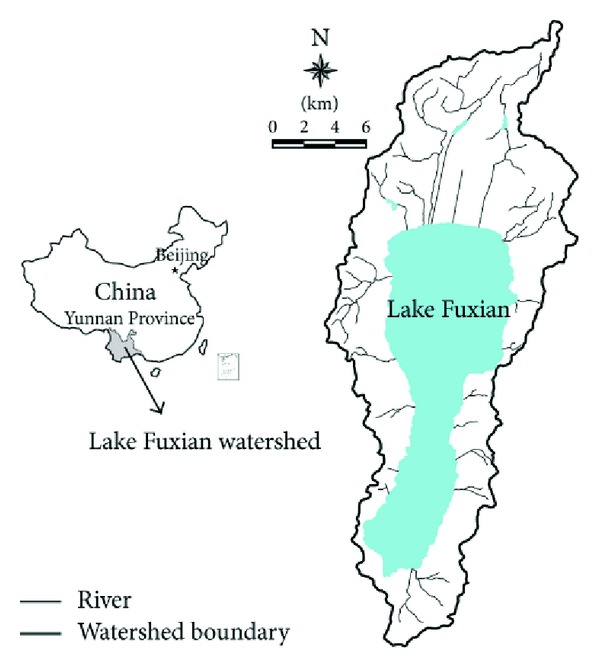
Location of Lake Fuxian watershed.

Lake Fuxian is located in Yuxi City, Yunnan Province, China (Figure 1). As the second deepest freshwater lake in China, Lake Fuxian has clear water, and its average water quality meets Class I of the China National Water Quality Standard [19]. The watershed is located between 24°21′28′′–24°38′00′′N and 102°49′12′′–102°57′26′′E with maximum lake depth of 151.5 m and average lake depth of 87 m. Lake Fuxian watershed covers an area of 674.69 km^2^ and has a volume of 20.55 billion m^3^, which amounts to approximately one tenth of Chinese freshwater storage volume. But the rivers flowing into Lake Fuxian have been severely polluted by farmland runoff, livestock husbandry, rural and urban life pollution and industrial production, and so on. Lake Fuxian is now facing the adverse transformation of water quality to Class II due to excessive watershed nutrient input. To harmonize water environment protection and economic development, it is essential to provide feasible industrial structure optimization schemes in Lake Fuxian watershed. 

### 2.2. REILP Approach

A typical ILP model can be formulated as follows [20]:

(1) $$\begin{array}{c} \text{max }f^{\pm }=C^{\pm }X^{\pm } \end{array}$$

subject to

(2) $$\begin{array}{c} A^{\pm }X^{\pm }\leq B^{\pm }, \end{array}$$

(3) $$\begin{array}{c} D^{\pm }X^{\pm }\geq E^{\pm }, \end{array}$$

(4) $$\begin{array}{c} X^{\pm }\geq 0, \end{array}$$

where ± is the interval upper and lower bound; *f* ^±^ is objective function or system return; *X* ^±^ = [*x*_1_^±^,*x*_2_^±^,…*x*_*n*_^±^]^*T*^ is the decision variable; *C* ^±^ = [*c*_1_^±^, *c*_2_^±^,…, *c*_*n*_^±^], *A* ^±^ = {*a*_*ij*_^±^}, and *D* ^±^ = {*d*_*ij*_^±^}  (*i* = 1,2,…*m*;  *j* = 1,2,…*n*) are model coefficients; *B* ^±^ = [*b*_1_^±^, *b*_2_^±^,…*b*_*m*_^±^]^*T*^ and *E* ^±^ = [*e*_1_^±^, *e*_2_^±^,…*e*_*m*_^±^]^*T*^ are constraint values.

Equations (1)–(3) can be spilt into the lower bound submodel and upper bound submodel by BWC (Best and Worst) algorithms [20]. The lower bound of objective function *f* ^−^ corresponding to the smallest decision risk can be obtained by the lower bound submodel, which is called “the most pessimistic condition.” The upper bound of objective function *f* ^+^ corresponding to the largest decision risk can be solved by the upper bound submodel, called “the most optimistic condition.”

To explore the trade-off relationship between system return and decision risk, a REILP model can be formulated as [17]

(5) min⁡ RISK  =⨁i{∑j=1n[λij(aij+−aij−)xj+γij(dij+−dij−)] +ηi(bi+−bi−)+κi(ei+−ei−)}subject  to  ∑j=1ncj−xj+λ0(cj+−cj−)xj≥f−+λ0(f+−f−),∑j=1naij+xj−bi−≤∑j=1nλij(aij+−aij−)xj+ηi(bi+−bi−), ∀i,∑j=1ndij−xj−ei++∑j=1nγij(dij+−dij−)+κi(ei+−ei−)≥0, ∀i,           X±≥0, ∀j,

where RISK represents the risk function of the entire system violating optimization model constraints; ⨁_*i*_ represents general arithmetic operator such as a simple or weighted addition, simple or weighted arithmetic mean; *λ*_*ij*_, *γ*_*ij*_, *η*_*i*_, *κ*_*i*_ are model variables, 0 ≤ *λ*_*ij*_, *γ*_*ij*_, *η*_*i*_, *κ*_*i*_ ≤ 1; *λ*_0_ represents the aspiration level determined by decision makers according to their risk preferences. 

The REILP model (5) can be solved by inputting different given discrete aspiration levels *λ*_0_. A higher aspiration level shows that decision makers expect to achieve better system return and could tolerate higher decision risk. When *λ*_0_ = 0, the REILP model is corresponding to the lower bound submodel of original ILP model. At this time the watershed system may achieve minimum system returns but have zero risk to violate constraint conditions. When *λ*_0_ = 1, the REILP model is corresponding to the upper bound submodel of original ILP model with maximum economic return and highest system risk.

**Figure 2:**
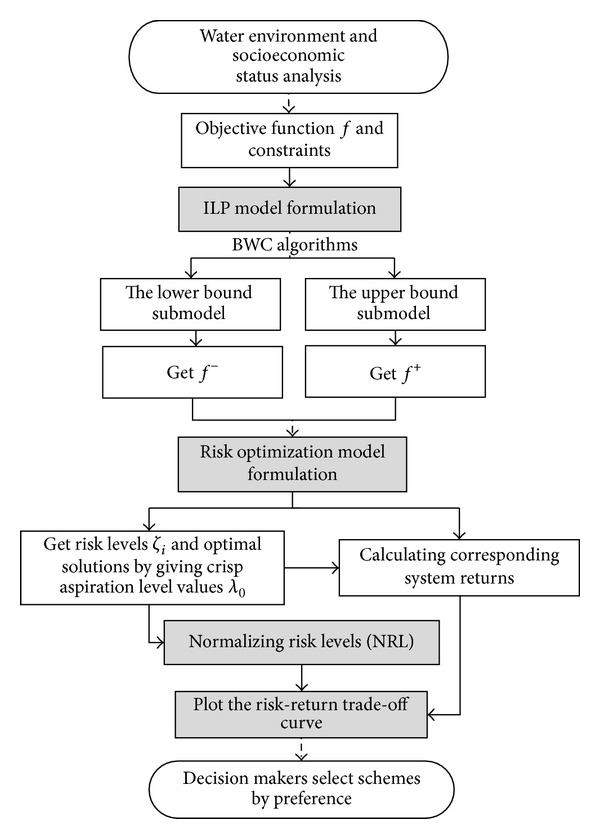
Solution steps of risk explicit interval linear programming model.

A complete REILP approach can be implemented through five steps altogether [17] (Figure 2): (1) formulate and solve an ILP model by BWC algorithms; (2) develop a risk optimization model; (3) solve the risk optimization model with different crisp aspiration level values *λ*_0_; (4) normalize the objective function of the risk optimization model to make normalized risk levels (NRL) equal to 0 in the most pessimistic condition and 1 in the most optimistic condition; (5) plot the risk-return trade-off curve to get the “low risk and high return efficiency” window and the “representative optimization schemes” at the turning point of the curve. 

## 3. Model Description and Formulation for Lake Fuxian Watershed

### 3.1. ILP Model

Alleviating the conflict between water environmental system and socioeconomic system was the key purpose of watershed optimization research in Lake Fuxian watershed. In 2009, farmland runoff pollution, phosphate mining point pollution, rural life sewage, feces and garbage pollution, livestock husbandry pollution, and dry and wet deposition were main nutrient load sources in Lake Fuxian watershed, which, respectively, accounted for 37.15%, 15.33%, 11.22%, 9.87%, and 15.49% of the total nitrogen (TN) pollution loads, as well as 19.69%, 22.34%, 13.57%, 16.76%, and 12.49% of the total phosphorus (TP) loads in Lake Fuxian. Considering the current pollution situation and economic development demand, this study focused on agriculture, forestry and livestock husbandry of the primary industry, phosphorus mining, and nonphosphorus industry of the secondary industry and tourism of the tertiary industry and selected corresponding industry scales as decision variables. The secondary industry in Lake Fuxian watershed depended mainly on phosphate mining and processing. There were 12 phosphorus chemical enterprises in the watershed which produced extremely serious phosphorus pollution. However, the phosphorus chemical enterprises were removed out of Lake Fuxian watershed in early 2010. So the secondary industry was classified into phosphorus chemical industry and nonphosphorus industry. For the local government, what was most expected was maximum economic benefit in the premise of protecting water quality and aquatic ecology. So the objective function of the ILP optimal model was developed to maximize the system returns, shown in (6).

The TP and TN loads into the lake were, respectively, beyond 58.3% and 24.0% of water environmental capacity with Class I in 2009. Load reduction was essential to maintain the water quality of Class I for Lake Fuxian. Therefore, environmental capacities for TP and TN were viewed as core constraints in the model (7)-(8). In recent years, the proportion of grain crops has dropped slowly and that of economic crops has risen gradually. But the amount of fertilizer applied in economic crops such as garlic and pea accounted for more than half of the total fertilizer dosage. Therefore, the areas of paddy, tobacco, garlic and pea, and flowers should be appropriately reduced, and the planting of organic vegetables needed to be largely popularized to protect water quality of Lake Fuxian. The crop farming structure was constrained as (9). Dominant small-scale captive way inevitably brought pollution to Lake Fuxian watershed with the largest proportion of pig breeding, followed by sheep. The number of cattle was relatively small with high nutrient emission coefficient. Adjusting livestock husbandry structure reasonably was very necessary in industrial structure optimization (10). 

The primeval vegetation in the Fuxian Lake watershed has been severely damaged, and the forest coverage rate was only 27.1% (excluding shrub land). Afforestation was in urgent need to increase forest cover rate and to moderate soil erosion (11). Resource constraints must also be considered for optimization strategies of industrial structure, including land area limitation, minimum farmland area required by the government, and water resources available restrictions (12)–(14). 

Lake Fuxian watershed had rather high proportion of primary industry, compared with 11.7% nationwide. Therefore inhibiting primary industry and encouraging the development of secondary and tertiary industries were essential to promote sustainable economic development. The specific constraints of three industries were shown in (15)–(17). The rural population should simultaneously migrate to the urban population to raise the level of urbanization because of the reduced primary industry (18). Lake Fuxian watershed had rich tourism resources and attracted millions of tourists for sightseeing annually. But tourism carrying capacity must be met though local government strongly encouraged the development of tourism (17). 

The ILP model for environmental economic optimization in Lake Fuxian watershed was shown as

(6) Max⁡f±=∑i=110NYj(EAGRi±)  SAGRijk±+∑i=1112NYj(INijk) +∑i=13NYj(ETOUi±)PTOUijk±, ∀j,k

subject to

(1) environmental capacity constraints:
  (i) TP environmental capacity constraints:
    (7) ∑i=110(RAGRPi±)SAGRijk±+∑i=1112(PINPi)INijk  +∑i=13(RTOUPi±)PTOUijk±  +∑i=1416(RRUWPi±)PRULjk±  +RURBP+RSOIP+RFALP≤PCAPk±, ∀j,k,
  (ii) TN environmental capacity constraints:
    (8) ∑i=110(RAGRNi±)SAGRijk±+∑i=1112(PINNi)INijk  +∑i=13(RTOUNi±)PTOUijk±+∑i=1416(RRUWNi±)PRULjk±  +RURBN+RSOIN+RFALN≤NCAP±, ∀j,k,
(2) farming structure constraints:
  (9) SEVEijkmin⁡≤SAGRijk±≤SEVEijkmax⁡, i=1,2,…,5;  ∀j,k,
(3) livestock husbandry structure constraints:
  (10) SEVEijkmin⁡≤SAGRijk±≤SEVEijkmax⁡, i=7,8,…,10;  ∀j,k,
(4) forest area constraints:
  (11) $$\begin{array}{c} \text{SAR}{\text{G}}_{6jk}^{\pm }\geq \text{SFO}{\text{R}}_{jk\min},\;\forall j,k, \end{array}$$
(5) resource constraints:
  (i) total land area constraints:
    (12) $$\begin{array}{c} \overset{6}{\underset{i=1}{\sum }}\text{SAG}{\text{R}}_{ijk}^{\pm }+\text{SLAk}+\text{STRA}\leq \text{STOL},\;\forall j,k, \end{array}$$
  (ii) arable land area constraints:
    (13) $$\begin{array}{c} \text{STE}{\text{P}}_{j\min}\leq \overset{5}{\underset{i=1}{\sum }}\text{SAG}{\text{R}}_{ijk}^{\pm }\leq \text{STE}{\text{P}}_{j\max},\;\forall j,k, \end{array}$$
  (iii) water resource constraints:
    (14) (WAGR)∑i=110SAGRijk±+(WIN)∑i=1112INijk+(WRUL)PRULjk±  +WURB≤WCAP, ∀j,k,
(6) industrial structure constraints:
  (i) agricultural output constraints:
    (15) $$\begin{array}{c} \overset{10}{\underset{i=1}{\sum }}(\text{EAG}{\text{R}}_{i}^{\pm })\text{SAG}{\text{R}}_{ijk}^{\pm }\geq \text{MAG}{\text{R}}_{jk}^{\pm },\;\forall j,k, \end{array}$$
  (ii) industrial output constraints:
    (16) $$\begin{array}{c} \text{SI}{\text{N}}_{jk\min}\leq \overset{12}{\underset{i=11}{\sum }}\text{I}{\text{N}}_{ijk}\leq \text{SI}{\text{N}}_{jk\max},\;\forall j,k, \end{array}$$
(7) tourism capacity constraints:
  (17) $$\begin{array}{c} \underset{i=13}{\sum }\text{PTO}{\text{U}}_{ijk}^{\pm }\leq \text{MTO}{\text{U}}_{j},\;\forall j,k, \end{array}$$
(8) rural population constraints:
  (18) $$\begin{array}{c} \text{PTO}{\text{L}}_{jk\min}\leq \text{PRU}{\text{L}}_{jk}^{\pm }\leq \text{PTO}{\text{L}}_{jk\max},\;\forall j,k, \end{array}$$
(9) technical constraints:
  (19) $$\begin{array}{c} \text{SAG}{\text{R}}_{ijk}^{\pm }\geq 0,\;\text{I}{\text{N}}_{ijk}\geq 0,\;\text{PTO}{\text{U}}_{ijk}^{\pm }\geq 0,\\ \text{PRU}{\text{L}}_{jk}^{\pm }\geq 0,\;\forall i,j,k, \end{array}$$

where *i* = 1,2,…, 13 represent 13 industry types, paddy, tobacco, garlic and pea, flowers, organic vegetables, woodland, cattle, pig, sheep, poultry, phosphate chemical industry, nonphosphate industry, and tourism, respectively; *j* = different optimization stages, *j* = 1,2, 3 for the 2010–2015, 2016–2020 and 2021–2030 periods, respectively; *k* = 1,2 for two scenarios; NY_*j*_ = the number of years during each stage; EAGR_*i*_^±^ = output value of unit farming industry or unit livestock husbandry ($10^4^/ha/yr or $10^4^/cattle/yr); SAGR_*ijk*_^±^ = area of different farming types (ha) or the number of different types of livestock (no); IN_*ijk*_ = industrial output ($10^4^); ETOU_*i*_^±^ = gains unit tourist ($/tourist/yr); PTOU_*ijk*_^±^ = number of tourists (10^4^ tourists); RAGRP_*i*_^±^, RAGRN_*i*_^±^ = TP or TN pollution coefficient of unit farming industry or unit livestock husbandry (kg/ha/yr or kg/cattle/yr); PINP_*i*_, PINN_*i*_ = TP or TN pollution coefficient of unit industrial output value (kg/$10^4^/yr); RTOUP_*i*_^±^, RTOUN_*i*_^±^ = TP or TN pollution coefficient of unit tourists (kg/tourist); RRUWP_*i*_^±^, RRUWN_*i*_^±^ = TP or TN pollution coefficient of rural domestic sewage for *i* = 1 or rural residents' excrement for *i* = 2 or rural domestic refuse for *i* = 3 (kg/10^4^ people/yr); PRUL_*jk*_^±^ = the number of rural residents (10^4^ people); RURBP, RURBN = TP or TN pollution coefficient of urban residents living (kg/yr); RSOIP, RSOIN = TP or TN pollution coefficient of soil erosion (kg/yr); RFALP, RFALN = TP or TN pollution coefficient of dry and wet deposition (kg/yr); PCAP_*k*_^±^, NCAP_*k*_^±^ = total phosphorus or nitrogen environment capacity (kg/yr); SEVE_*ijk*max⁡_, SEVE_*ijk*min⁡_ = the maximum or minimum area of each farming type (ha); NEVE_*ijk*max⁡_, NEVE_*ijk*min⁡_ = the maximum or minimum number of each livestock (no); SFOR_*jk*min⁡_ = minimum area of woodland (ha); SLAk = surface area of Lake Fuxian (ha); STRA = traffic area (ha); STOL = the total area of Lake Fuxian watershed (ha); STEP_*j*max⁡_, STEP_*j*min⁡_ = the maximum or minimum arable land (ha); WAGR = water demand of unit arable land or unit livestock husbandry output value (m^3^/ha/yr or m^3^/$10^4^/yr); WIN = water demand of unit industrial output (m^3^/$10^4^/yr); WRUL = rural water demand per capita rural people (m^3^/10^4^ people/yr); WURB = water demand of urban life (m^3^/yr); WCAP = annual dynamic water available (m^3^/yr); MAGR_*jk*_^±^ = minimum agriculture output value ($10^4^); SIN_*jk*max⁡_, SIN_*jk*min⁡_ = the highest and lowest output value of industry ($10^4^); MTOU_*j*_ = tourism carrying capacity (10^4^ people); PTOL_*jk*max⁡_, PTOL_*jk*min⁡_ = the maximum or minimum number of rural population (10^4^ people). All parameters data for objective function and constraints were obtained from Yuxi statistical yearbooks [21] and relevant research report [22]. The primary model parameters are listed in Table 1.

### 3.2. REILP Model

To explore the trade-offs between decision risk and system return to provide feasible optimization schemes for decision makers, a REILP model was developed for environmental economic optimization in Lake Fuxian watershed based on (5):

(20) $$\begin{array}{c} \text{min RISK}=\overset{3}{\underset{n=1}{\sum }}\text{RIS}{\text{K}}_{n}, \end{array}$$

where *n* is the number of the constraints whose coefficients are interval numbers in the ILP model; RISK_*n*_ is the risk function of each constraint. Detailed expressions were shown as follows:

(21) RISK1=[∑i=110(RAGRPi+−RAGRPi−)(SAGRijk)λijk +∑i=13(RTOUPi+−RTOUPi−)(PTOUijk)λijk  +∑i=1416(RRUWPi+−RRUWPi−)(PRULjk)λijk +λ17jk(PCAPk+−PCAPk−)]×(PCAPk−)−1, ∀j,k,RISK2=[∑i=110(RAGRNi+−RAGRNi−)(SAGRijk)γijk   +∑i=13(RTOUNi+−RTOUNi−)(PTOUijk)γijk   +∑i=1416(RRUWNi+−RRUWNi−)(PRULjk)γijk   +  γ17jk(NCAPk+−NCAPk−)]×(NCAPk−)−1, ∀j,k,RISK3=[∑i=110(EAGRi+−EAGRi−)(SAGRijk)ρijk   +ρ11jk(MAGRjk+−MAGRjk−)]×(MAGRjk−)−1,                          ∀j,k

subject to

(22) ∑i=110[EAGRi−+(EAGRi+−EAGRi−)λ0]SAGRijk  +∑i=1112INijk+∑i=13[ETOUi−+(ETOUi+−ETOUi−)λ0]  ×PTOUijk≥fjk−+(fjk+−fjk−)λ0, ∀j,k,∑i=110(RAGRPi+)SAGRijk+∑i=13(RTOUPi+)PTOUijk  +∑i=1416(RRUWPi+)PRULjk−PCAPk− ≤∑i=110(RAGRPi+−RAGRPi−)(SAGRijk)λijk  +∑i=13(RTOUPi+−RTOUPi−)(PTOUijk)λijk  +∑i=1416(RRUWPi+−RRUWPi−)(PRULjk)λijk  +λ17jk(PCAPk+−PCAPk−), ∀j,k,∑i=110(RAGRNi+)SAGRijk+∑i=13(RTOUNi+)PTOUijk  +∑i=1416(RRUWNi+)PRULjk−NCAPk− ≤∑i=110(RAGRNi+−RAGRNi−)(SAGRijk)γijk  +∑i=13(RTOUNi+−RTOUNi−)(PTOUijk)γijk  +∑i=1416(RRUWNi+−RRUWNi−)(PRULjk)γijk  +γ17jk(NCAPk+−NCAPk−), ∀j,k,∑i=110(EAGRi+−EAGRi−)(SAGRijk)ρijk  +ρ11jk(MAGRjk+−MAGRjk−) ≥∑i=16(EAGRi−)SAGRijk  +∑i=710(ESTOi−)NSTOijk−MAGRjk+, ∀j,k,0≤λijk,γijk,ρijk≤1.

The other constraints and model parameter values of the REILP model were exactly the same as (9)–(14) and (16)–(19) in the ILP model. LINGO is an interactive and general optimization solver which can conveniently and effectively build and solve the linear, nonlinear, and integer optimization model. Simple equation description, powerful solvers, and fast execution speed of LINGO are so attractive that the REILP model in the Lake Fuxian watershed was solved by the LINGO 12.0 software to get optimization schemes.

## 4. Results and Discussion

### 4.1. Scenario Setting

Scenario analysis was introduced into the solution process of ILP optimal model to ensure the practicality and operability of alternative optimization schemes [23]. Two scenarios were set in this study. Scenario I focused on coordinating environmental protection and economic growth. The right-hand value in the TP environmental capacity constraints was equal to water environment capacity of Class I. But Lake Fuxian may not maintain water quality standard of Class I according to the constraints of Scenario I since model ignored the dynamic, complexity, and ecological vulnerability of the watershed system. Thus, Scenario II incorporated interactive processes in an attempt to reach more stringent constraints on water quality protection. That was to say TP loads into Lake Fuxian were less than 90% of the environmental capacity. The interval ranges in some other constraints were loosened appropriately to meet the requirements of environmental sustainability. For example, the lower bounds for severely polluted industry became smaller, such as crop farming and livestock husbandry; the upper bounds for organic vegetables with less nutrient pollution were larger than Scenario I in Period III, and the minimum constraint of woodland area had also declined slightly.

This study made short-, medium-, and long-term planning with the base year of 2009, and the model was solved for three periods, 2010–2015, 2016–2020, and 2021–2030, respectively. All parameters involved in the model were based on field investigations, previous research reports, statistical yearbooks, and local agencies. The specific constraints in three stages under two scenarios were shown in Table 2.

### 4.2. Results of ILP Model

The system returns of ILP model were shown in Table 3 by solving lower bound submodels and upper bound submodels. The lower bound of system returns meant minimum total system returns with the smallest decision risk, which corresponded to “the most pessimistic condition.” On the other hand, “the most optimistic condition” for the upper bound of system returns had maximum economic benefits and highest system risk. Through comparative analysis between two scenarios, we could find that Scenario II had smaller lower bound values, but larger upper bound values in every optimization period. This was because some constraints under Scenario II were appropriately loosened to allow system returns to have more extensive interval ranges though constraint on TP load was stricter.

### 4.3. Risk Level-System Return Analysis and Discussion

**Figure 3:**
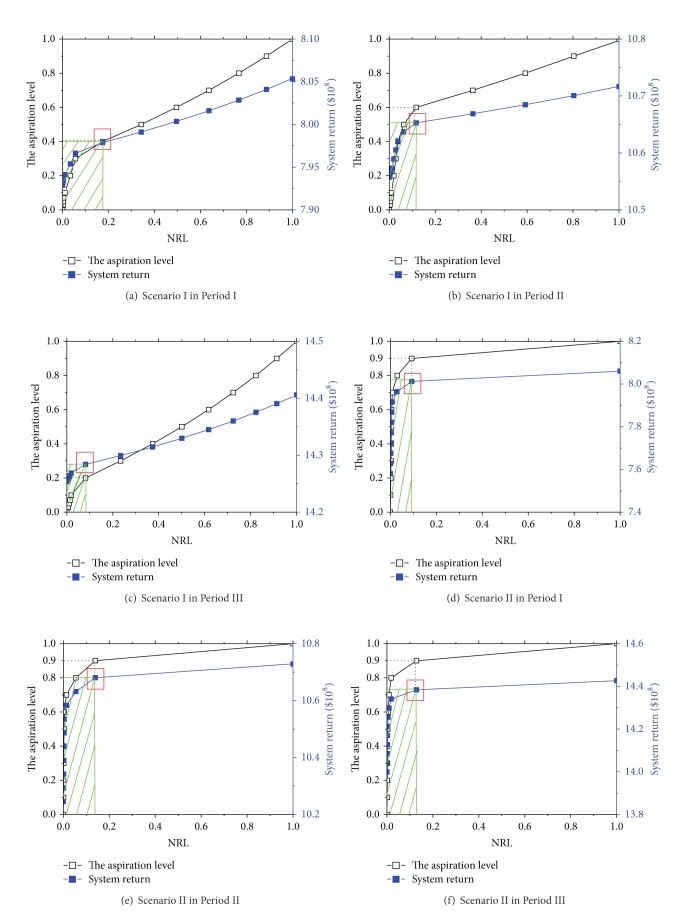
Trade-off curve for three planning periods under two scenarios.

To further explore the trade-off relationship between system return and decision risk, the REILP model could be solved based on specific constraints in three stages under two scenarios (Table 2) and the objective function values of ILP model (Table 3) by inputting different crisp aspiration level values. The results of system returns, normalized risk levels, and industrial structure optimization schemes were shown in Table 4 for Period I under Scenario I and Table 5 for Period II under Scenario II. Decision makers may explicitly select optimization schemes of industrial structure according to their expected system returns and tolerant decision risks. Table 4 showed that most of the decision variable values (labeled with the symbol “∗”) happened to change when 0.20 ≤ *λ*_0_ ≤ 0.40 (0.00 < NRL < 0.20), while the decision variable values of Table 5 changed at 0.80 ≤ *λ*_0_ ≤ 1.00 (0.00 < NRL < 0.20), which was consistent with the corresponding aspiration level of the turning point of trade-off curve (or NRL-system return curve, Figure 3). The difference of *λ*_0_ corresponding to changes of decision variables mainly resulted from inconsistent TP load constraints and some industrial structure constraints to produce different feasible solution domains between two scenarios.

Figure 3 described the trade-off relationship between watershed total system returns and normalized risk levels. The aspiration level of *λ*_0_ was an essential input variable to get uncertainty risk and system return by solving the model, and NRLs linked the aspiration level and system return as an intermediate variable. So when we knew the tolerable uncertainty risk of decision makers, system return and *λ*_0_ could be directly got by NRL-system return relationship (the blue line with closed dots) and NRL-the aspiration level curve (the black line with opened dots), respectively. Then solve the REILP model to obtain industrial structure optimization scheme with the known *λ*_0_. When the aspiration level selected by decision makers was 0, there was no decision risk. At this time, the optimization scheme of industrial structure had the most conservative perception of system uncertainty but could only gain the least economic benefits. In contrast, when *λ*_0_ was input into the value 1.00, decision makers had the most risky optimization scheme to gain the most system returns, which amounted to the results of upper bound submodel in ILP model. 

For Scenario I (Figures 3(a)–3(c)), system return increased sharply when the NRL gradually rose from 0.00 to 0.08~0.18 (the value was different for different periods). This indicated that a significant increasing speed of system benefits could be achieved under small risk. The window of “low risk and high return efficiency” (outlined by slash) was most desired for decision makers who were willing to take a small risk of watershed management decisions. When the NRL continued to rise above 0.08~0.10, system return was still increasing but a slower speed than before. This window could only gain low return efficiency increasing unit risk, which was called “high risk and low return efficiency.” The optimization schemes in this window were unfavorable for decision makers to carry out unless they were willing to accept unobvious benefit increasing and take a high risk of management decisions. 

The two windows were more contrasting in Scenario II (Figures 3(d)–3(f)). The system return was almost constant when the NRL rose above 0.15, and this strongly highlighted the advantages of the window of “low risk and high return efficiency” (outlined by slash). Thus, the scheme at the turning point (marked by rectangle with red line) was the most representative in most watershed management cases. The study clearly advised decision makers to give priority to the representative scheme with relatively small risk of system uncertainty and almost highest income in practical optimization decisions.

### 4.4. Analysis of the Representative Scheme

From trade-off curves (Figure 3), we could find the aspiration level *λ*_0_ corresponding to y-coordinates of turning points in the NRL-the aspiration level curve. Then input *λ*_0_ into the NRL-system return relationship to get the representative optimization schemes. To better understand the industrial structure optimization schemes in Lake Fuxian watershed and comparatively analyze the two scenarios at six turning points, the representative schemes of *λ*_0_ = 0.40, *λ*_0_ = 0.60, *λ*_0_ = 0.20 in three periods under Scenario I and *λ*_0_ = 0.90 in three periods under Scenario II were further analyzed and compared.

#### 4.4.1. Industrial Structure Optimization

**Figure 4:**
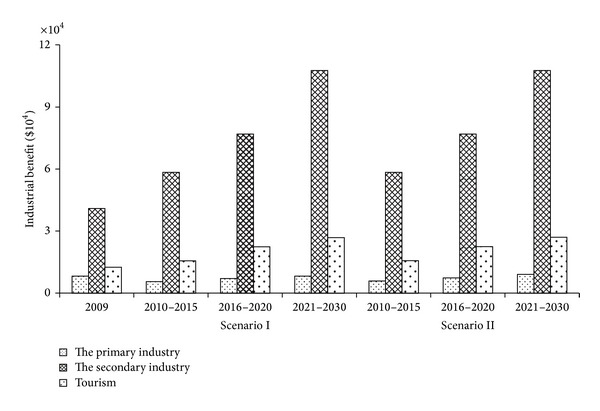
Three-industry structure of the representative optimization scheme.

Figure 4 showed that the average annual output values of the primary industry, the secondary industry, and tourism had been rising in three planning stages to meet macroeconomic development need in Lake Fuxian watershed. From the perspective of strategic planning, the total annual output value of three industries had increased nearly 30% of the base year in Period I, almost 73% in Period II, and more than one time in Period III. The rapid economic development was most desired under the premise of water quality protection in watershed water environmental-economic system management decisions.

The comparative analysis of two scenarios could find that every industry output values in Scenario II were almost the same as or even slightly higher than Scenario I. And Scenario II had more stringent requirements of TP loads into the lake which were essential to protect ecological system health of Lake Fuxian watershed. High economic return and low system risk fully embodied the superiority of Scenario II.

#### 4.4.2. The Primary Industrial Structure Optimization

**Figure 5:**
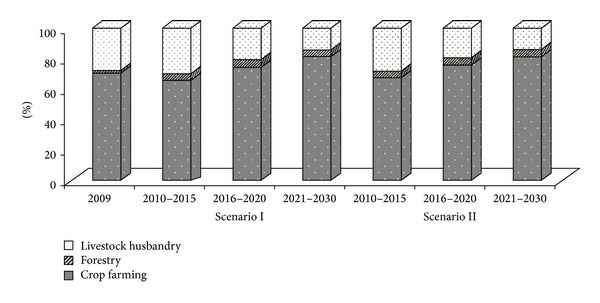
Proportion of crop farming, forestry, and livestock husbandry benefits.

Livestock husbandry was in a declining trend in the representative optimization scheme (Figure 5) which was very beneficial for load reduction of livestock pollution. In order to raise the forest cover ratio, forestry output value had been rising in Lake Fuxian watershed, but the proportion in the primary industry was in a stable condition during the entire planning period. The proportion of farming in Period I was lower than the base year of 2009 because of a significant reduction in acreage of high pollution crops such as paddy, tobacco, garlic, pea, and flowers. However, little pollution crops such as organic vegetables would be widely promoted and planted in Period II and Period III to bring very high benefit. The proportion of crop farming in primary industry was still great and represented the upward trend throughout the entire planning stage. From the view of crop farming output value, forestry, and livestock husbandry, Scenario II was prior to Scenario I. 

#### 4.4.3. Farming Structure Optimization

**Figure 6:**
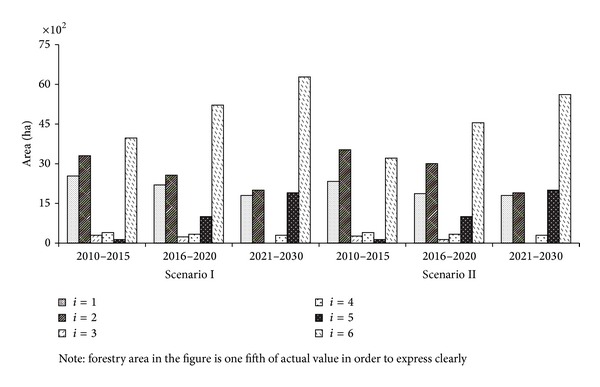
Planting types optimization results.

To control nonpoint source pollution fully in Lake Fuxian watershed, the planting area of paddy, tobacco, garlic, pea, and flowers was declining all the time in three planning stages (Figure 6). Among them, fertilization quantity of garlic and vegetable pea was almost two times of other crops. Therefore, Lake Fuxian watershed no longer grew garlic and vegetable pea in Period III which was replaced by a massive planting of organic vegetables.

Every crop area in Scenario II was less than or equaled to that in Scenario I except flue-cured tobacco and organic vegetables in three periods. This indicated Scenario II was more beneficial to lower the TP and TN pollution loads and protect water quality of Lake Fuxian. But from the perspective of forest coverage and soil loss prevention, Scenario II was slightly inferior to Scenario I because of the smaller forest area.

#### 4.4.4. Livestock Husbandry Structure Optimization

**Figure 7:**
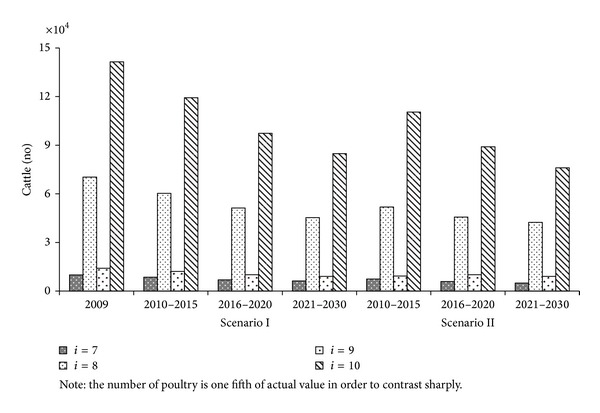
Livestock husbandry optimization results.

Figure 7 showed the optimization results of livestock husbandry structure at the representative turning points of trade-off curves in Period I, Period II and Period III under two scenarios. The number of each type of livestock had been declining from 2010 to 2030. For example, the number of pigs under Scenario I had reduced by 14.22%, 27.02%, and 35.56%, respectively, in the three periods. Scenario II had a higher reduction proportion than Scenario I because TP environmental capacity was strictly constrained for the same period.

#### 4.4.5. Pollutant Loads into the Lake Distribution

The primary industrial pollution and secondary industrial pollution were major pollution sources of Lake Fuxian watershed in the base year (Table 6). Reasonable structure adjustment of farming and livestock husbandry resulted in a substantial reduction of primary industry pollution loads into the lake in three stages. But the TN and TP loads into Lake Fuxian still came mainly from the primary industrial pollution and rural life pollution. All phosphorus companies were removed out of Lake Fuxian watershed in early 2010, which would make secondary industrial pollution loads close to zero. Lake Fuxian watershed was encouraged to develop the leisure and ecocultural tourism industry, so the number of tourists would be rising within the planning stages. At the same time, the pollution loads generated by the tourism industry had increased slightly, but the total amount was not large.

TN and TP loads into Lake Fuxian in Scenario II were lower for corresponded planning stage since Scenario II had smaller acreage of most crops and smaller number of livestock than Scenario I. This coincided with the stringent requirements of TP load into the lake less than 90% of environment capacity. In summary, the optimization scheme of Scenario II was believed to be more advantageous which was most desirable for policy makers to make industrial structure optimization in Lake Fuxian watershed. 

## 5. Conclusions

A traditional ILP model may lead to invalid optimization scheme and infeasible decision support, so the REILP approach was applied to overcome the shortages and explore the trade-offs between decision risk and system return under uncertainty. The industrial structure of Lake Fuxian watershed was extremely unreasonable, and its water quality was adversely transforming from Class I to II. This study had developed a risk explicit interval linear programming model for uncertainty-based environmental economic optimization in Lake Fuxian watershed. It was rather convenient for decision makers to incorporate the trade-off information into their considerations for water environment and socioeconomic system optimization implementation plans. After analysis of risk level-system return curve, the window of “low risk and high return efficiency” should be most considered for decision makers because of its large benefit increasing rate. Especially the optimization schemes at the turning points had absolute advantages of relatively low risk and nearly maximum gains and had guiding significance for achieving sustainable development of Lake Fuxian watershed. Through scenario analysis and comparison discussions, the study found that the representative scheme under Scenario II was preferable to Scenario I whether from the perspective of economic return or considering water quality protection. Decision makers could develop an efficient and practical industrial structure optimization scheme based directly on the REILP solution.

Risk explicit interval linear programming model is a rather practical and effective tool to promote the harmonious development of environment and economy. However, it should be noted that a few processes were cursorily considered for lack of enough data in Lake Fuxian watershed. For example, TN and TP pollution coefficients from urban residents living, soil erosion, and dry and wet deposition were all treated as a constant value during the whole programming periods. Therefore, the optimization results may not be quite coincident with the real situation, and the realistic sense of calculated values was limited. Since the main object of this study is to provide new insights to make environmental economic optimization for watershed with unreasonable industrial structure, our study could provide the right direction and scientific reference for decision makers in Lake Fuxian watershed.

## Figures and Tables

**Table 1:** Parameter values of ILP model.

	*i *	EAGR_*i*_^±^	RAGRN_*i*_^±^	RAGRP_*i*_^±^
Primary industry	1	[0.138, 0.162]	[6.870, 10.995]	[0.510, 0.810]
	2	[0.432, 0.524]	[28.575, 45.705]	[0.765, 1.230]
	3	[0.450, 0.542]	[15.315, 24.510]	[1.275, 2.040]
	4	[3.212, 3.305]	[12.255, 19.605]	[1.020, 1.635]
	5	[2.379, 2.610]	[1.605, 2.565]	[0.135, 0.210]
	6	[0.010, 0.016]	[4.410, 7.350]	[0.675, 1.125]
	7	[0.011, 0.014]	[4.888, 6.110]	[0.806, 1.007]
	8	[0.015, 0.018]	[0.361, 0.451]	[0.136, 0.170]
	9	[0.002, 0.005]	[0.182, 0.228]	[0.036, 0.045]
	10	[0.0008, 0.0011]	[0.022, 0.028]	[0.009, 0.012]

	*i *		PINN_*i*_	PINP_*i*_

Secondary industry	11	—	[48.425, 48.425]	[11.115, 11.115]
	12	—	[0.016, 0.016]	[0.000, 0.000]

	*i*	ETOU_*i*_^±^	RTOUN_*i*_^±^	RTOUP_*i*_^±^

Tertiary industry	13	[44.737, 45.045]	[7.170, 11.472]	[0.965, 1.544]

**Table 2:** Constraints in three periods under two scenarios.

	*i *	Scenario I			Scenario II		
		Period I	Period II	Period III	Period I	Period II	Period III
Agriculture (10^4^ ha)	1	0.25–0.33	0.22–0.25	0.18–0.22	0.23–0.33	0.19–0.25	0.18–0.22
	2	0.30–0.37	0.20–0.33	0.20–0.23	0.27–0.37	0.19–0.28	0.17–0.23
	3	0.027–0.030	0.013–0.0.023	0	0.027–0.030	0.013–0.023	0
	4	0.03-0.04	0.02-0.03	0.02-0.03	0.03-0.04	0.02-0.03	0.02-0.03
	5	0.00-0.01	0.05–0.10	0.10–0.13	0.00-0.01	0.05–0.10	0.10-0.20
	6	1.94–2.47	2.61–3.47	3.14–4.14	1.61–2.47	2.27–3.47	3.14–4.14
	*∑*	0.60–0.67	0.57–0.63	0.53–0.60	0.53–0.67	0.50–0.65	0.53–0.60

Livestock husbandry (10^4^ no)	7	0.833–0.853	0.673–0.713	0.578–0.623	0.743–0.853	0.583–0.713	0.488–0.623
	8	5.811–6.031	4.811–5.131	4.163–4.531	5.181–6.031	4.081–5.131	3.431–4.531
	9	1.188–1.208	0.968–1.008	0.838–0.908	0.928–1.208	0.708–1.008	0.578–0.908
	10	59.60–59.70	48.50–48.70	42.00–42.40	55.20–59.70	44.50–48.70	38.00–42.40

Output value ($10^6^)	Phosphate industry	0	0	0	0	0	0
	Nonphosphate industry	461.5–584.6	615.4–769.2	923.1–1076.9	415.4–584.6	615.4–769.2	923.1–1076.9
	Primary industry	49.2–∞	61.5–∞	80.0–∞	38.5–∞	53.9–∞	69.2–∞

Tourism	(10^4^ tourists)	300–350	400–500	500–600	290–350	400–500	500–600

Rural people	(10^4^ people)	10.7–12.0	10.0–11.5	8.5–10.0	10.7–12.0	10.0–11.5	8.5–10.0

Environmental carrying capacity (ton)	TN	940.6–994.8	940.6–994.8	940.6–994.8	846.54–895.30	846.54–895.30	846.54–895.30
	TP	115.8–122.67	115.8–122.67	115.8–122.67	104.22–110.40	104.22–110.40	104.22–110.40

**Table 3:** The system returns of ILP model.

System return ($10^4^)	Base year	Scenario I			Scenario II		
		Period I	Period II	Period III	Period I	Period II
The lower bound	61776.02	79289.22	105573.95	142535.18	75803.48	102450.23	139987.75
The upper bound	61776.02	80534.09	107166.11	144059.08	80606.55	107286.88	144267.69

**Table 4:** Risk explicit analysis in Period I under Scenario I.

Decision variable	Unit	Scenario I in Period I					
		*λ*_0_ = 0.00	*λ*_0_ = 0.20	*λ*_0_ = 0.40	*λ*_0_ = 0.60	*λ*_0_ = 0.80	*λ*_0_ = 1.00
System return	$10^4^	79289.22	79538.19	79787.17	80036.14	80285.12	80534.09
NRL	—	0.000	0.033	0.174	0.495	0.767	1.000
*i* = 1	ha	2533.3	2533.3	2533.3	2533.3	2533.3	2533.3
*i* = 2	ha	3307.6*	3300.3*	3300.0	3300.0	3300.0	3300.0
*i* = 3	ha	266.7	266.7*	300.0*	300.0	300.0	300.0
*i* = 4	ha	400.0	400.0	400.0	400.0	400.0	400.0
*i* = 5	ha	133.3	133.3	133.3	133.3	133.3	133.3
*i* = 6	ha	19400.7	19400.7*	19849.6*	21749.0*	23534.6*	24734.0*
*i* = 7	no	8331	8331*	8531*	8531	8531	8531
*i* = 8	no	58113*	59232.05*	60313*	60313	60313	60313
*i* = 9	no	11883	11883*	12083*	12083	12083	12083
*i* = 10	no	596038	596038*	597038*	597038	597038	597038
*i* = 11	$10^4^	0	0	0	0	0	0
*i* = 12	$10^4^	58461.54	58461.54	58461.54	58461.54	58461.54	58461.54
*i* = 13	10^4^ tourists	350	350	350	350	350	350
Rural people	10^4^ people	10.7	10.7	10.7	10.7	10.7	10.7

Note: *denotes changing variables.

**Table 5:** Risk explicit analysis in Period II under Scenario II.

Decision variable	Unit	Scenario II in Period II					
		*λ*_0_ = 0.00	*λ*_0_ = 0.20	*λ*_0_ = 0.40	*λ*_0_ = 0.60	*λ*_0_ = 0.80	*λ*_0_ = 1.00
System return	$10^4^	102450.23	103417.56	104384.89	105352.22	106319.55	107286.88
NRL	—	0.000	0.001	0.003	0.004	0.055	1.000
*i* = 1	ha	1866.7	1866.7	1866.7	1866.7	1866.7	1866.7
*i* = 2	ha	1866.7	1866.7	1866.7	1866.7*	2474.0*	2900.0*
*i* = 3	ha	133.3	133.3	133.3	133.3	133.3	233.3
*i* = 4	ha	200.0	200.0	200.0	200.0*	333.3*	333.3
*i* = 5	ha	980.3	980.3	980.3*	996.3*	1000.0*	1000.0
*i* = 6	ha	22734.0	22734.0	22734.0	22734.0	22734.0	34734.0
*i* = 7	no	5831	5831	5831	5831	5831*	7131*
*i* = 8	no	40813	40813	40813	40813	40813*	50313*
*i* = 9	no	7083	7083	7083	7083	7083*	10083*
*i* = 10	no	445038	445038	445038	445038	445038*	487038*
*i* = 11	$10^4^	0	0	0	0	0	0
*i* = 12	$10^4^	76923.08	76923.08	76923.08	76923.08	76923.08	76923.08
*i* = 13	10^4^ tourists	450*	466.66*	483.0312*	499.3575*	500*	500
Rural people	10^4^ people	10	10	10	10	10	10

Note: *denotes changing variables.

**Table 6:** TP and TN loads distribution of representative scheme.

Industry types (ton)	Nutrient kinds	Base year	Scenario I			Scenario II		
			Period I	Period II	Period III	Period I	Period II	Period III
The primary industry	TP	76.98	39.03	38.85	39.58	33.95	36.67	35.40
	TN	614.57	277.59	272.36	282.72	265.81	263.11	246.47

Industry	TP	40.98	0.03	0.04	0.06	0.03	0.04	0.06
	TN	179.33	0.91	1.20	1.68	0.91	1.20	1.68

Tourism	TP	0.38	0.34	0.48	0.58	0.34	0.48	0.58
	TN	2.82	2.51	3.59	4.30	2.51	3.59	4.30

Rural life	TP	24.87	14.56	13.60	11.56	14.56	13.60	11.56
	TN	130.87	80.05	74.81	63.59	80.05	74.81	63.59

Total	TP	143.21	53.96	52.97	51.72	48.88	50.79	47.60
	TN	927.59	361.06	351.96	352.29	349.28	342.71	316.04
